# Supplementary material for: Electrophysiological mechanisms of human memory consolidation
Source: Nat Commun. 2018 Oct 5;9:4103. doi: 10.1038/s41467-018-06553-y (PMC6173724; doi:10.1038/s41467-018-06553-y)
Supplement: Supplementary file 1 — Supplementary Information [file 41467_2018_6553_MOESM1_ESM.pdf]

Supplementary Information

Electrophysiological mechanisms of human memory consolidation

H. Zhang et al

Supplementary Notes

Supplementary Note 1. Behavioral results

Participants remembered 43±19% (mean±STD) of all remote items and 48±22% of all recent items (*t(11)*=2.15, *p*=0.054). These hit rates were higher than false alarm rates (33±18%) for both remote (*t(11)*=4.77, *p*=0.00058) and recent items (*t(11)*=4.14, *p*=0.0016). We compared (a) remote and recent images and (b) remembered and forgotten images with regard to color and intensity (the size of all images in the study is the same, namely 640 x 480 pixels). Color and intensity were assessed by averaging the RGB values (separately for R, G, and B) and intensity values across pixels and then compared between the different conditions. We did not find that (a) remote and recent items or (b) remembered and forgotten items differed with regard to color or intensity (all t<1.79, all p>0.10).

Stimulus-specific representations

Supplementary Note 2. Evaluate frequencies of interest

To identify EEG frequencies showing item-specific representations, we performed a frequency-resolved representational similarity analysis similar to Zhang et al [1]. Data preprocessing was the same as in the main data analysis, including trial segmentation, time-frequency transformation, and z-transformation of EEG power within each channel and each frequency. Instead of averaging the EEG power within conventional frequency bands, we kept the individual frequencies (1 to 200Hz with 1Hz as step). Thus, we obtained a time x frequency x channel (13 x 200 x channel) power matrix separately for each trial during encoding and retrieval. The frequencies of interest were identified by representational similarity analysis.

First, we calculated a non-parametric Spearman's correlation between encoding of a picture (*n* pictures after artifact rejection, with $n \leqslant 320$, which is the total number of pictures during the remote and recent sessions across both days) and retrieval of either the same picture (RSA_{same}) or a different picture (RSA_{differ}). Correlations were calculated across electrodes, separately for each time window and each frequency. For each patient, this yielded a time x frequency x *n* similarity matrix of RSA_{same} values (with time = 13 being the number of time windows during encoding and retrieval; *frequency* = 200 being the number of frequencies from 1Hz to 200Hz with 1Hz as step), and a time x frequency x *n* x (*n-1*) matrix of RSA_{differ} values. Second, the Fisher-Z transformed correlation matrices were averaged across trials for both RSA_{same} and RSA_{differ}, separately for each patient. This generated two 13 x 200 (time x frequency) similarity matrixes – one for RSA_{same} and one for RSA_{differ} – for each patient. Third, we compared RSA_{same} and RSA_{differ} matrices using paired t-tests across patients within each time-frequency bin. Fourth, we also compared RSA_{same} values against 0 using a one sample t-test across patients within each time-frequency bin. This was done to ensure that RSA_{same} values were reliably larger than zero.

Statistical results of multiple time-frequency bins were corrected for multiple comparisons using surrogate based cluster statistics. First, we extracted cluster values from the empirical data (i.e., based

on the actual difference between RSA_{same} and RSA_{differ} matrices and between RSA_{same} and 0): We thresholded the t-value of each time-frequency bin such that only bins with t-values corresponding to p-values smaller than 0.01 in both comparisons were taken into account. Time bins with shared edges were defined as belonging to the same cluster. T-values (RSA_{same} vs. RSA_{differ}) of each contiguous cluster of *time x frequency* values were summed up. These empirical cluster values were compared to a distribution of surrogate clusters. To obtain these surrogate clusters, we randomly shuffled trial labels (within each patient and frequency band) and performed the same RSA procedure as described above. We extracted clusters from these surrogate matrices and selected the cluster with the largest summary t-value (RSA_{same} vs. RSA_{differ}) for each surrogate. This surrogate procedure was repeated 10,000 times and produced 10,000 surrogate clusters (if no cluster was found in one of the surrogates, a cluster value of 0 was assumed for this surrogate). Then, we ranked each empirical cluster within the distribution of all surrogate clusters. Clusters with summary t-values larger than 99% (alpha level = .01) of all surrogate t-clusters were selected. We found a cluster showing item-specific representations ranging from 35Hz to 130Hz (Supplementary Fig. 2a) which was largely overlapping with the frequency range reported in previous papers [1, 2].

In the current project, we focused on two frequency bands, the gamma band (30-90Hz) and the epsilon band (90-150Hz) as defined previously (e.g. [3]). We then assessed the contribution of different frequency ranges to functionally relevant stimulus-specific representations during encoding in greater detail. We found that functionally relevant stimulus-specific representations occurred only at frequencies up to 90Hz but not above: In detail, during encoding, we investigated stimulus-specific representations within four sub-bands between 30 and 150Hz (30-60Hz, 60-90Hz, 90-120Hz, and 120-150Hz). We applied the same RSA approach as reported for the main analysis. Within each sub-band, we correlated the distributed EEG power between encoding and retrieval of the same item across electrodes and compared it with correlation values between encoding and retrieval of different items. As indicated in Supplementary Fig. 2b we found functionally relevant stimulus-specific clusters within the 30-60Hz and 60-90Hz bands but not within the sub-bands above 90Hz (90-120Hz and 120-150Hz) (by performing a paired t-test between RSA values of remembered vs. forgotten items across participants within each cluster). Notably, we also found functionally relevant stimulus-specific representations when excluding activity from the ripple band, i.e. when focusing on 30-80Hz activity.

Supplementary Note 3. Stimulus-specific representations at higher time resolution

We first extracted for each channel and each item the iEEG activity within the gamma band (30-90Hz) and used consecutive time windows of 100ms, overlapping by 50ms. The temporal resolution was twice as high as the one reported in our main data. We then applied the same analysis as described in Fig. 1c, i.e. analyzing encoding-retrieval similarity of same and different items in different time windows. Consistent with the results reported in the main paper, we observed clusters showing significantly higher correlation between encoding and retrieval of the same items compared with different items. As illustrated in Supplementary Fig. 2c, three separate clusters were identified: 650ms-1,200ms, 150ms-550ms, and 850-1,000ms. Again, we found that activity during later, but not earlier time windows during

encoding was behaviorally relevant for later retrieval, because encoding-retrieval similarity was higher for remembered than forgotten items in the late (650-1,200ms) cluster ($t(11)$=2.78, p=0.018) but not the other two clusters ($t(11)$<1.01, p>0.34). This result is highly consistent with the results reported in the main paper, which were based on consecutive time windows of 200ms, overlapping by 100ms.

Supplementary Note 4. Autocorrelation effects

We estimated the amount of temporal autocorrelations in distributed representations at gamma power (30-90Hz). First, we correlated 30-90Hz EEG power between any two time bins of each encoding trial across electrodes (using time bins of 10ms). This analysis results in an $n \times n \times m$ matrix for each participant (n: number of time bins in each trial; m: number of trials). Second, Fisher-Z-transformed correlation values were averaged across trials by matching the temporal distance between two correlated time bins. Correlations corresponding to a temporal distance of 0 were discarded. This generated a $1 \times (n\text{-}1)$ vector for each participant. Finally, we compared the correlation value at each temporal distance with the average correlation value across all temporal distances by performing a paired T-test across participants. We found that the autocorrelation was significantly above the average only during intervals of up to 250 milliseconds (Supplementary Fig. 2e). Autocorrelations between bins with intervals above 250 milliseconds did not differ from the averaged value. This result is consistent with results from a similar analysis in our previous study [1] showing that the temporal autocorrelation of intracranial EEG data decays quickly, in particular at high frequencies. Since autocorrelations are restricted to such short time intervals, they do not affect correlations between encoding and retrieval or between encoding and resting state.

Spontaneous replay

Supplementary Note 5. Replay of gamma activity during the early encoding cluster

We assessed replay of gamma band activity from the early encoding cluster (100-500ms) showing stimulus-specific representations that were not related to subsequent memory. We found that replay levels were significantly greater than zero for remote items ($t(11)$=2.28, p=0.043), but not for recent items ($t(11)$=0.76, p=0.47). Replay of activity from this early cluster was marginally different between remote and recent items ($t(11)$=2.19, p=0.051; Supplementary Fig. 4a). When analyzed separately for the different states of vigilance, replay of remote items was greater than zero during nREM sleep ($t(9)$=3.10, p=0.012), but not during waking state ($t(11)$=1.66, p=0.12) and not for recent items (waking state: $t(11)$=0.17, p=0.87; nREM sleep: $t(9)$=0.39, p=0.70). Furthermore, replay levels of remote items were not significantly higher than those of recent items (waking state: $t(11)$=1.44, p=0.18; nREM sleep: $t(9)$=2.09, p=0.066). We did not find different replay levels between waking state and nREM sleep for remote items ($t(9)$=1.80, p=0.11).

Supplementary Note 6. Replay of gamma band activity sequences

Rodent research has provided evidence not only for replay of individual spatial locations by single place cells, but also for replay of sequences of place cells representing the trajectory that the animal has travelled before [4, 5, 6]. Indeed, several theories of hippocampal function assume a specific role for memory of temporal sequences [7, 8, 9]. We thus investigated replay not only for activity during individual encoding time windows, but also for the sequence of activity across consecutive encoding time windows. We created electrode x sequence matrices during the early and the late encoding time window showing stimulus-specific activity, and concatenated them into vectors (Supplementary Fig. 5a). For the late encoding cluster (500-1,200ms), we found that replay levels were significantly greater than zero for remote items ($t(11)$=3.16, p=0.0091), but not for recent items ($t(11)$=0.56, p=0.59; Supplementary Fig. 5b). Replay was higher for remote items compared with recent items ($t(11)$=2.35, p=0.038). Replay of remote items was larger than zero during both nREM sleep ($t(9)$=2.88, p=0.018) and waking state ($t(11)$=2.76, p=0.019). Replay levels of remote items were higher than those of recent items during waking state ($t(11)$=2.38, p=0.037) but there was only a trend for an increase during nREM sleep ($t(9)$=2.17, p=0.058). The direct comparison between remembered and forgotten remote items was not significant ($t(11)$=0.47, p=0.65).

Analysis of the early encoding cluster showed similar results, but less pronounced (Supplementary Fig. 5c). Replay levels of remote items were marginally larger than zero ($t(11)$=2.19, p=0.051) and were marginally higher than replay levels of recent items (recent vs. zero: $t(11)$=0.56, p=0.59; remote vs. recent: $t(11)$=2.10, p=0.059). When analyzed separately for the different states of vigilance, replay of remote items was greater than zero during nREM sleep ($t(9)$=2.71, p=0.024), but not during waking state ($t(11)$=1.70, p=0.12) and not for recent items (waking state: $t(11)$=0.12, p=0.91; nREM sleep: $t(9)$=0.24, p=0.81). Replay levels of remote items were not significantly higher than those of recent items (waking state: $t(11)$=1.86, p=0.090; nREM sleep: $t(9)$=2.00, p=0.076). Replay levels did not differ between waking state and nREM sleep for remote items for either cluster (both $t(9)$<1.94, p>0.84). In general, the results were very similar to replay of individual encoding time windows reported in the main text and in Supplementary Note 5.

Supplementary Note 7. Replay of clusters in the epsilon band

Replay of epsilon band activity during the two clusters (300-700ms and 500-900ms) showing stimulus-specific representations was evaluated using the same approach as used for the two clusters in the gamma band. However, activity during rest was not more strongly correlated with activity during remote items as compared to recent items for either cluster, either across the whole rest period (all $t(11)$<1.41, all p>0.18) or within the different states of vigilance (all $t(11)$<1.85, all p>0.091). Replay levels did not differ between waking state and nREM sleep within either cluster for remote items (both $t(9)$<1.21, p>0.25). Thus, activity from the two encoding clusters in the epsilon band showing stimulus-specific representations was not spontaneously replayed.

Supplementary Note 8. Replay of activity sequences in the epsilon band

Analogous to the analysis of gamma band activity, we also assessed whether epsilon band activity sequences were replayed. For both encoding clusters showing stimulus-specific representations (300-700ms and 500-900ms), replay of activity was not different between remote and recent items (300-700ms: $t(11)$=1.68, p=0.12; 500-900ms: $t(11)$=2.18, p=0.052; Supplementary Fig. 5d, e). When analyzing different states of vigilance separately, we did not find higher replay of remote than recent items either (waking state: 300-700ms: $t(11)$=2.06, p=0.064; 500-900ms: $t(11)$=2.14, p=0.056; nREM sleep: 300-700ms: $t(9)$=1.55, p=0.15; 500-900ms: $t(9)$=1.79, p=0.11). Replay levels of remote items did not differ between waking state and nREM sleep for either cluster (both $t(9)$<1.19, p>0.26).

Supplementary Note 9. Temporally-compressed or expanded replay in the gamma band

We also considered possible temporally compressed (or expanded) replay by using the following scale parameter (scale parameter = encoding time length/rest time length): 0.2, 0.4, 0.6, 0.8, 1.2, 1.4, 1.6, 1.8, and 2.0. We found that the replay level of remote items was higher than the replay level of recent items with all scale parameters for the late encoding cluster (all $t(11)$>2.31, all p<0.044). We did not find any difference between temporally-compressed/expanded replay to replay levels at the original temporal scale (all $t(11)$<0.89, p>0.38). Since our data show substantial temporal autocorrelation within intervals of up to 250ms (Supplementary Note 4; Supplementary Fig. 2e), we believe that these results need to be interpreted with caution and should be addressed in future studies.

Relationship of spontaneous replay to gamma power

Supplementary Note 10. Replay and gamma power during encoding

Averaged EEG power in the gamma frequency range (30-90Hz) was higher for remote as compared to recent items during the two encoding time clusters showing stimulus-specific representations (500-1,200ms: $t(11)$=2.54, p=0.028; 100-500ms: $t(11)$=2.35, p=0.038). We thus needed to rule out that different replay levels of remote and recent items were related to gamma power during encoding. We performed two control analyses. First, we ranked all recent and remote items according to their gamma power during the two clusters showing stimulus-specific representations (late cluster: 500-1,200ms; early cluster: 100-500ms) within each patient. We then gradually removed trials with extreme power values for both remote and recent items until the averaged gamma power of recent items was comparable to or even slightly higher than the averaged gamma power of remote items for each patient. For example, if initially the averaged gamma power of recent items was smaller than the averaged gamma power of remote items, we removed the recent item with the least gamma power and the remote item with the most gamma power. This procedure was then repeated until the averaged gamma power of recent items was equal or slightly higher than the averaged gamma power of remote items (comparison of gamma power between recent and remote items after trial removal: late cluster: $t(11)$=

-2.20, p=0.0497 [higher gamma power for recent than remote items]; early cluster: $t(11)$=-1.30, p=0.22). For these selected trials, replay of remote items was still significantly higher than replay of recent items (late cluster: $t(11)$=2.39, p=0.036; see also Supplementary Fig. 6a; early cluster: $t(11)$=2.58, p=0.026). As a second control analysis, we correlated gamma power during encoding with replay levels across all items, separately within each patient. Gamma power was extracted from the same encoding clusters as described above. Correlations were calculated separately for each patient, and then the Fisher-Z transformed correlation coefficients across all patients were compared to zero. We found no evidence for any consistent correlations (late cluster: $t(11)$=-0.14, p=0.89; early cluster: $t(11)$=0.51, p=0.62; Supplementary Fig. 6b). Together, these results show that replay is not related to gamma power during encoding.

Supplementary Note 11. Replay and gamma power during rest

We next assessed if replay levels were related to the power of gamma band activity (30-90Hz) during the rest period. First, we averaged the replay levels across the two encoding clusters showing stimulus-specific representations (late cluster: 500-1,200ms; early cluster: 100-500ms) and across all trials within each rest time window (lasting 200ms with 100ms overlap). Second, we averaged the gamma power (30-90Hz) within each rest window. Third, we correlated the averaged replay levels and EEG power across all rest time windows using non-parametric Spearman's correlations. The Fisher-Z transformed correlation coefficients were on average numerically lower than zero, reminiscent of the negative relationship between gamma power and stimulus-specific representations found in a previous study [1]. However, the correlation coefficients were not consistently different from zero for either the late or the early cluster (both $t(11)$>-1.60, both p>0.14; Supplementary Fig. 6c). These results show that replay levels are unrelated to the magnitude of gamma power during the rest period.

Supplementary Note 12. Temporal proximity effects

In our experimental design, the temporal distances between rest periods and items presented in the remote and recent conditions are similar. This design should effectively control for any possible biases induced by temporal autocorrelations. To empirically exclude any possible effect of temporal proximity on replay, we fitted a linear regression line to all replay values over the whole rest period for both the late (500-1,200ms) and the early (100-500ms) encoding cluster, separately for all remote and recent items. We averaged the slope of these regressions across all remote and recent items in each patient and then performed a t-test on the slope of these linear regressions against zero across patients. A temporal proximity effect should result in a negative slope of replay values across rest for the remote condition (higher replay at the beginning of the rest period, lower replay at later rest time windows), and a positive slope for recent items. However, slope values did not differ from zero for either the late or the early cluster and either remote or recent items (all $t(11)$<1.58, all p>0.14; Supplementary Fig. 6d). These results indicate that the temporal proximity or distance between encoding of an item and a specific time interval during rest does not affect replay levels.

Ripple-triggered replay

Supplementary Note 13. Ripple-triggered replay vs. spontaneous replay

To further investigate the enhanced ripple-locked replay during nREM sleep, we performed a comparison between ripple-locked replay and spontaneous replay. Because ripples are relatively rare events as compared to the overall duration of the resting state (duration of ripple events vs. overall duration of the resting state = 1:292 ± 74; mean±SD), we matched the duration and incidence between ripple events and rest epochs within each patient. First, we computed ripple-locked replay levels of the late encoding cluster (showing functionally relevant stimulus-specific representations) during nREM sleep for remembered remote items for each participant and performed a one-sample t-test against zero across participants. This resulted in a t-value (t_{ripple}). We then compared t_{ripple} to a surrogate distribution of t-values using the same contrast but applied to randomly selected epochs. These epochs were selected by matching the duration and number of ripple events within each participant. We then calculated the replay levels within these randomly selected epochs for the late encoding cluster of remembered remote items within each patient and, again, performed a one-sample t-test against zero across participants. This again resulted in a t-value ($t_{\text{spontaneous}}$). We repeated the procedure of randomly selecting matched epochs for 10,000 times, resulting in 10,000 $t_{\text{spontaneous}}$ values. We then ranked t_{ripple} within the distribution of $t_{\text{spontaneous}}$ values. We found that t_{ripple} was higher than 95.47% of values of $t_{\text{spontaneous}}$ (corresponding to p=0.0453), consistent with the result showing that the replay level of late encoding activities of remembered remote items during ripple events was higher than replay levels during peri-ripple periods during nREM sleep.

We also performed the same analysis for ripple-triggered replay of activity from the early encoding time cluster for forgotten remote items during NREM sleep and found a significantly higher replay as compared to the distribution of $t_{\text{spontaneous}}$ values (p=0.0157), again corroborating our results showing that the replay level of early encoding activities of forgotten remote items during ripple events was higher than replay levels during peri-ripple periods during nREM sleep.

By contrast, when we conducted the same analysis for replay of late encoding activity of forgotten items, for replay of early encoding activity of remembered items, or for replay during waking state, we did not find any significant effects (all t_{ripple} smaller than 58.06% of $t_{\text{spontaneous}}$). Ripple-locked replay of activity from the late encoding cluster of forgotten remote items during nREM sleep was even smaller than spontaneous replay levels (t_{ripple} smaller than 98.6% of $t_{\text{spontaneous}}$, corresponding to p=0.014).

We also directly compared replay levels during ripple events with spontaneous replay levels of the entire rest periods after segmenting the entire resting period into many small epochs lasting as long as the average duration of ripple events within each participant, excluding periods containing ripple events. Again, we first analyzed replay of activity from the late encoding activities for remembered items during nREM sleep. Replay levels during ripples and outside of ripples were averaged in each participant. We then performed a paired T-test across participants between ripple-locked replay and spontaneous replay. This generated an empirical t-value which we then compared to surrogate t-values. Due to the

large difference of the number of data between ripple-locked replay and spontaneous replay, we estimated the significance level of the empirical t-value using surrogate t-values to rule out any effects caused by the largely different amount of data. Within each permutation, we collapsed ripple-locked replay and spontaneous replay within each participant and then randomly drew the same number of data as surrogate "ripple events". We performed the same paired T-test across participants, resulting in a surrogate t-value. This surrogate procedure was repeated 10,000 times, resulting in 10,000 surrogate t-values. We then ranked the empirical t-value within the distribution of surrogate t-values. We found that the empirical t value was higher than 94.99% of surrogate t-values, corresponding to p=0.0501. We applied the same analysis to the late encoding activities for forgotten items during nREM sleep and both remembered and forgotten items during waking state and the early encoding activities for both remembered and forgotten items during both waking and nREM sleep. We found that the ripple-locked relay level of the early encoding activities was higher than the spontaneous replay level for forgotten items during nREM sleep (p=0.0037). No other results were significant (all p>0.11).

Supplementary Note 14. Ripple-triggered vs. surrogate ripple-triggered replay

Surrogate ripple events were extracted from channels other than the hippocampal channel that was used for detection of ripple events. For each iteration of the surrogate extraction, we randomly selected one channel for each participant and extracted surrogate ripple events following the same procedure as for the empirical ripple extraction. The EEG power (80-100Hz) of surrogate ripple events did not differ from the power of hippocampal ripple events ($t(11)$=1.31, p=0.22). In line with our main finding on spontaneous replay, we found that surrogate-ripple-locked replay levels of both the early and late encoding activities were higher for remote than recent items both for remembered and forgotten items locked to surrogate ripples (all $t(9)$>3.02, all p<0.012). Importantly, however, when we compared replay during surrogate ripple events with surrogate peri-ripple periods during nREM sleep, we did not find any difference for either the early or the late encoding activities, either for remembered or forgotten remote items (all $t(9)$<1.16, all p>0.13).

We also directly compared replay of remote items during empirical (hippocampal) ripple events with replay levels during surrogate ripple events for the late encoding activities of remembered remote items during nREM sleep. We first focused on replay of activity from the late encoding cluster during ripple events and compared it against zero by performing a one-sample T-test across participants. This generated one empirical $t_{\text{empirical}}$ value, which was then compared to the distribution of 10,000 surrogate $t_{\text{surrogate}}$ values. For remembered items during nREM sleep, we found that the $t_{\text{empirical}}$ value was higher than 95.23% (corresponding to p=0.0477) of the $t_{\text{surrogate}}$ values for the late encoding activities (500-1,200ms). For forgotten items, we observed a trend for replay of activity from the early encoding cluster (p=0.0697), similar to our result of enhanced replay during ripple events as compared to peri-ripple periods. We performed the same analysis for replay of late encoding activities of forgotten items during nREM sleep and for replay of early and late encoding activities of remembered and forgotten items during waking state and did not find any significant results (all p>0.1031).

Supplementary Note 15. Ripple-triggered replay of remote items during waking state

As illustrated in Fig. 4c, replay levels of remote items were only enhanced during ripple events in nREM sleep, but not during waking state. We quantified this by performing the same analysis as applied to ripples in nREM sleep on replay of activity from the early (100-500ms) and late encoding cluster (500-1,200ms; Fig. 4c-d) for both remembered and forgotten items by performing a 2 X 2 way ANOVA and found no interaction effect ($F(1,10)$=0.24, p=0.64). For the late encoding cluster (500-1,200ms), replay levels did not differ between ripple events and peri-ripple periods for either later remembered ($t(10)$=0.28, p=0.78; Supplementary Fig. 8a) or later forgotten items ($t(10)$=0.78, p=0.45). We then performed the same analysis for activity from the early encoding cluster (100-500ms). Again, replay levels did not differ between ripple events and peri-ripple periods for either later remembered ($t(10)$=0.66, p=0.52) or later forgotten items ($t(10)$=0.43, p=0.68). Finally, we compared replay levels between ripple events and peri-ripple periods for all separate encoding time windows, but again did not find any difference. Even though we did not find a contribution of ripples during awake resting state to replay, this may be explained by considering that the brain is more dynamic during waking state and therefore replay may be more difficult to detect.

Supplementary Note 16. Ripple-triggered replay of recent items

Replay levels of later remembered and forgotten recent items during different vigilance states are depicted in Supplementary Fig. 8c. Unlike the enhanced replay levels of remembered remote items during ripple events in nREM sleep, we did not observe similar effects for recent items, either in nREM sleep or during waking state. First, we did not find an interaction between the early and late cluster for remembered and forgotten items for either nREM sleep ($F(1,9)$=2.62, p=0.14) or waking state ($F(1,9)$=0.0047, p=0.95) for recent items. Second, we analyzed the replay level of activity from the late encoding cluster (500-1,200ms) for both later remembered and forgotten recent items, separately for waking state and nREM sleep. We did not find any difference of replay levels between ripple events and peri-ripple periods for either nREM sleep (remembered: $t(9)$=0.007, p=0.99; forgotten: $t(9)$=0.66, p=0.52) or waking state (remembered: $t(10)$=1.83, p=0.094; forgotten: $t(10)$=0.62, p=0.55). Third, we also did not find any difference of replay levels of activity from the early encoding cluster (100-500ms) during either nREM sleep (remembered: $t(9)$=1.55, p=0.16; forgotten: $t(9)$=0.72, p=0.49) or waking state (remembered: $t(10)$=0.41, p=0.69; forgotten: $t(10)$=0.59, p=0.57).

Relationship between ripple-triggered replay and EEG power

Supplementary Note 17. EEG power during waking state and nREM sleep ripples

We extracted the ripple events from different states of vigilance (waking state and nREM sleep). We found that the EEG power at hippocampal electrodes in the ripple frequency range (80-100Hz) during

ripple events was higher for ripples during nREM sleep than during waking state (*t(8)*=6.05, *p*<0.001; Supplementary Fig. 7). To rule out that these power differences may account for the different ripple-locked replay patterns between the two different states of vigilance, we performed a control analysis. We extracted ripple events with the highest ripple power during waking state, until the power difference between the two states of vigilance was no longer significant (*t(8)*=1.06, *p*=0.32). Still, waking state replay levels of remote items did not differ between ripple events and peri-ripple periods, for either remembered or forgotten items, either during the late (500-1,200ms) or the early (100-500ms) encoding cluster (all (*t(10)*<1.28, all *p*>0.22). These results rule out that the different ripple-locked replay patterns during waking and nREM stages are due to the ripple power difference between waking state and nREM sleep.

Supplementary Note 18. EEG power during ripple events and peri-ripple periods

We next analyzed if the power of gamma band activity (30-90Hz) during ripple events or peri-ripple periods was related to different replay levels during nREM sleep. We averaged EEG power across the gamma band separately across ripple events and peri-ripple periods within each hippocampus electrode which was selected for ripple extraction. Gamma power was higher during ripple events as compared to peri-ripple periods (paired T-tests: *t(9)*=11.65, *p*<0.001). This may have been expected because ripples were defined via power maxima between 80-100Hz.

In the analysis of spontaneous replay, we found that replay levels were not correlated with gamma power during rest (see above, Supplementary Note 11 and Supplementary Fig. 6c). Thus, the fact that gamma power increased during ripple events (as compared to peri-ripple periods) per se cannot explain the increased replay levels during ripples – in particular considering that replay levels were specifically increased during nREM sleep ripples for remote items but not for recent items and also not for remote or recent items during waking state ripples.

To further rule out any effect of gamma power during ripple events on replay levels, we conducted two control analyses. First, we extracted the hippocampal gamma power (30-90Hz) during the ripple period and the averaged gamma power during peri-ripple periods for each ripple event. Then, we averaged the replay levels across all items from both the remote and recent sessions for the early and the late clusters separately during each ripple event and each (averaged) peri-ripple period. Then we correlated the gamma power and the replay level across ripple and peri-ripple periods for both the early and the late cluster within each patient. The Fisher-Z-transformed correlation values did not differ consistently from zero across patients for either the early (*t(9)*=1.75, all *p*=0.11) or the late cluster (*t(9)*=1.51, all *p*=0.17). As a second control analysis, we selected epochs with elevated gamma power during rest satisfying two criteria: 1) the gamma power (30-90Hz) from electrodes in the hippocampus had to rank higher than 99% of the entire rest period; 2) the ripple power (80-100Hz) from the same electrode had to rank below the top 1% of the entire rest period (i.e., not fulfilling the criterion for a ripple event). By directly comparing replay levels of these selected epochs with replay levels within five epochs before and after the selected epochs, we found no difference for either the early or the late encoding activities during

either nREM sleep or waking state for either remembered or forgotten items (all $t<1.41$, $p>0.20$). No interaction effect between clusters (early vs. late encoding activities) and memory performance (remembered vs. forgotten items) was found during either nREM sleep or waking state (both $F<0.50$, $p>0.62$). These results show that high replay levels of remembered items during nREM sleep ripple events are not due to hippocampal gamma power.

Brain regions	# of contacts
Frontal Cortex	16
Occipital Cortex	2
Parietal Cortex	2
Medial Temporal Lobe	104
Lateral Temporal Cortex	75

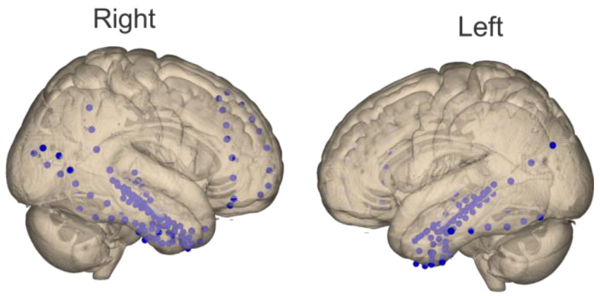

Supplementary Fig. 1. Distribution of electrode contacts across the brain. Only electrode contacts contralateral to the epileptic zone and without relevant contamination by epileptiform activity are shown.

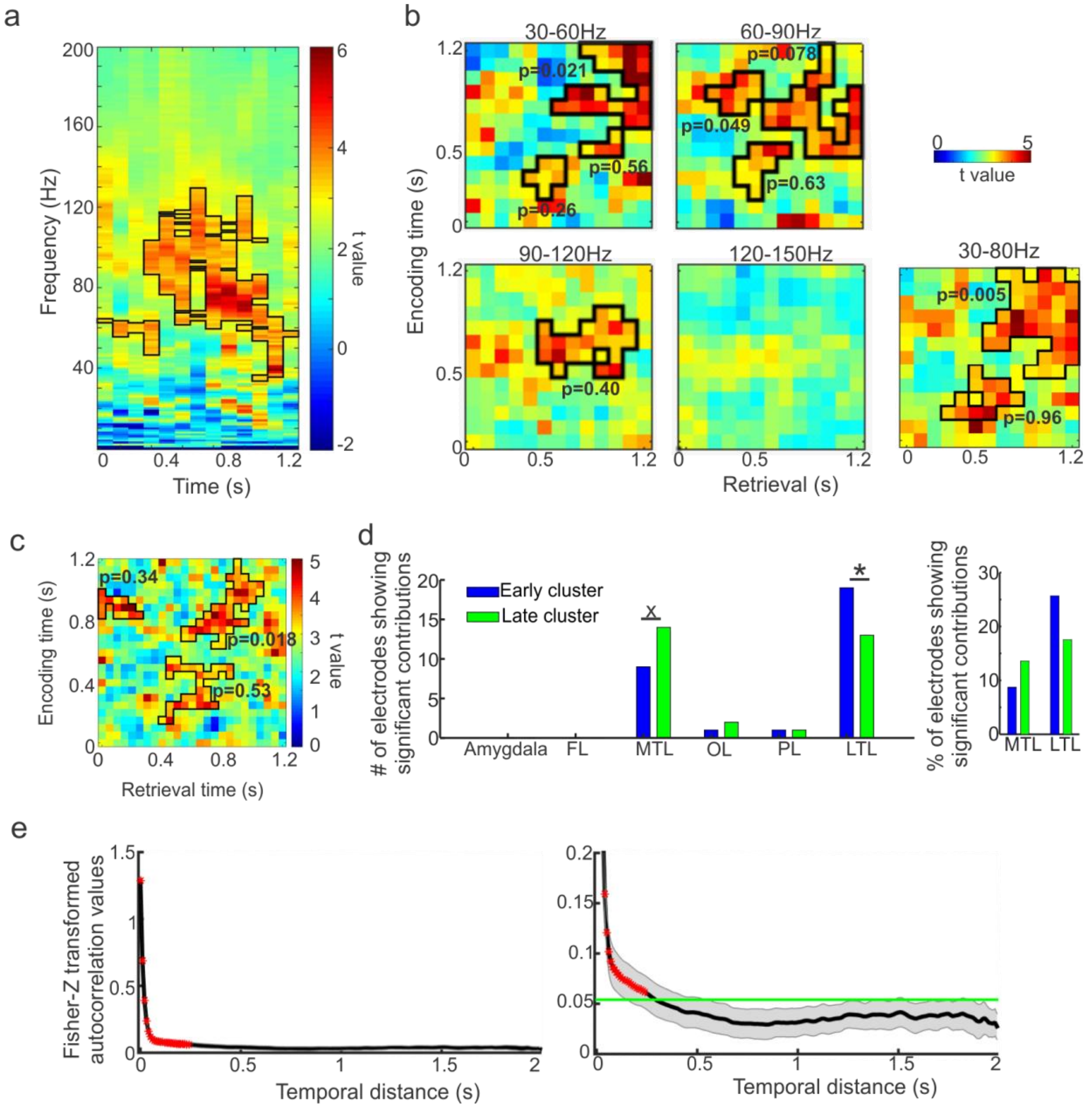

Supplementary Fig. 2. Identification of frequency bands of interest and assessment of temporal autocorrelations. (a) Clusters showing stimulus-specific representations from 35Hz to 130Hz. **(b)** Clusters showing stimulus-specific representations (i.e., higher correlations between encoding of one and retrieval of the same item as compared to encoding of one and retrieval of a different item) for four different sub-bands between 30-150Hz and for a gamma band between 30-80Hz. P-values beside each cluster indicate the results of assessing the functional relevance of stimulus-specific representations for memory (comparing RSA values between remembered and forgotten items within each cluster). **(c)** Three clusters showed stimulus-specific representations (i.e., higher correlations between encoding of one and retrieval of the same item as compared to encoding of one and retrieval of a different item) in the gamma range using a high temporal resolution with consecutive time window of 100ms, overlapping by 50ms. P-values beside each cluster indicate the results of assessing the functional relevance of stimulus-specific representations for memory (comparing RSA values between remembered and forgotten items within each cluster). **(d)** Left panel: Distribution of electrodes showing significantly positive contribution of stimulus-specific representations of the early and the late clusters. Right panel:

Percentage of electrodes showing positive contribution to the early and the late encoding clusters in MTL and lateral temporal lobe. FL: frontal lobe; PL: parietal lobe; OL: occipital lobe; LTL: lateral temporal lobe; MTL: medial temporal lobe; x indicates $p=0.085$; * indicates $p<0.05$ (Chi-square test). **(e)** Assessment of autocorrelations. The right panel is a magnification of the left panel. Red *s indicate correlations higher than the mean correlations. The green line indicates mean correlation values.

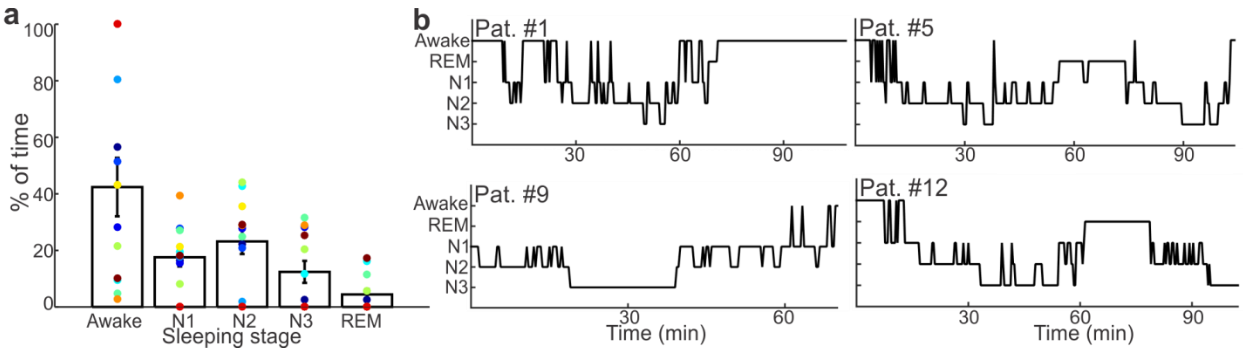

Supplementary Fig. 3. Sleep staging. (a) Proportion of time spent in the different states of vigilance. Error bars indicate standard errors of the mean (SEM). Each color dot indicates one patient; dots colored the same correspond to the same patient. **(b)** Example hypnograms of 4 patients.

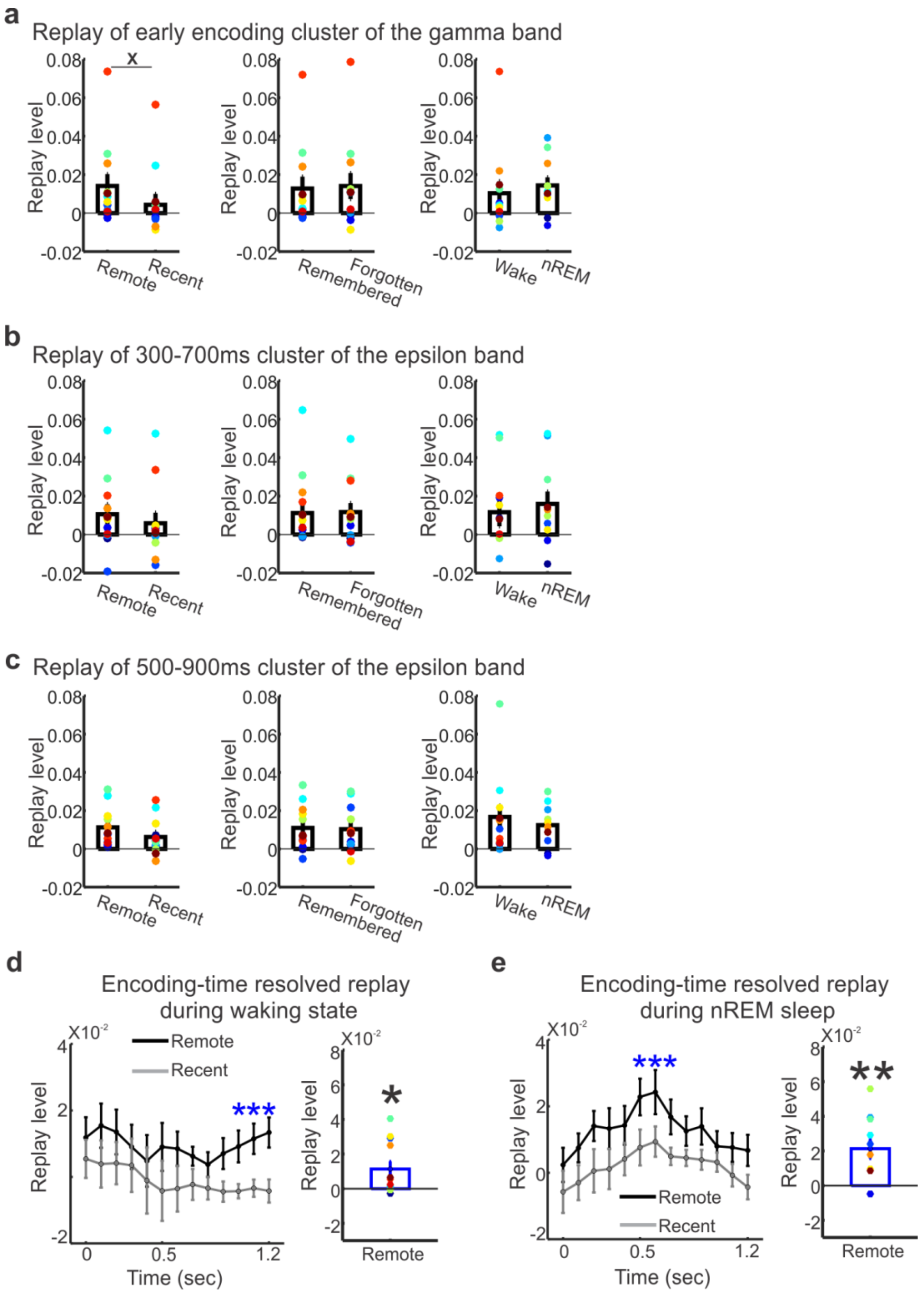

Supplementary Fig. 4 Additional results on spontaneous replay. Spontaneous replay levels of the early encoding cluster (100-500ms) of gamma band activity **(a)** and in the 300-700ms **(b)** and the 500-900ms **(c)** cluster of epsilon band activity. Encoding-time resolved replay during waking state **(d)** and nREM sleep **(e)** of gamma band activity. Error bars indicate standard error of the mean (SEM); each color dot indicates one patient; * indicates $p < 0.05$ (paired T-test in left panels of d and e; one-sample T-test in right panels of d and e); ** indicates $p < 0.01$ (one-sample T-test); (x) indicates p=0.051 (paired T-test).

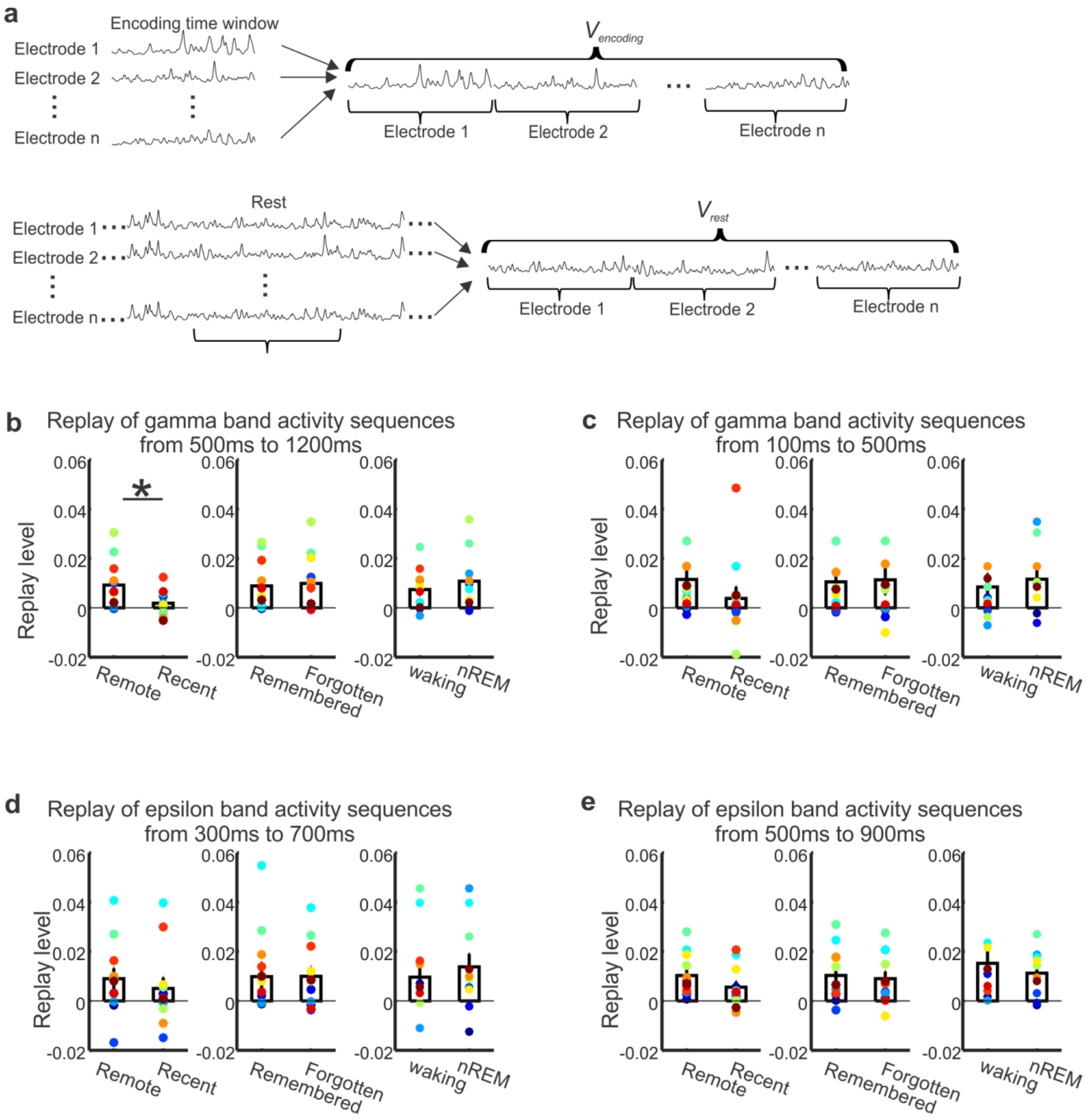

Supplementary Fig. 5. Spontaneous replay of encoding-related activity sequences. (a) Schema of extracting $V_{encoding}$ and V_{rest} to estimate the spontaneous replay of activity sequences during encoding within the two clusters showing stimulus-specific representations. The gamma band activity sequences during the late (500-1,200) but not the early (100-500ms) encoding time window show higher replay levels for remote items than for recent items (**b,c** left panels). Replay levels do not differ between remembered and forgotten remote items (**b,c** middle panels) or between waking state and nREM sleep (**b,c** right panels). The epsilon band activity sequences during either cluster did not differ between remote and recent items (**d,e** left panels), between remembered and forgotten items (**d,e** middle panels) or between waking state and nREM sleep (**d,e** right panels). Error bars indicate standard error of the mean (SEM); each color dot indicates one patient; dots colored the same correspond to the same patient; * indicates $p < 0.05$ (paired T-test).

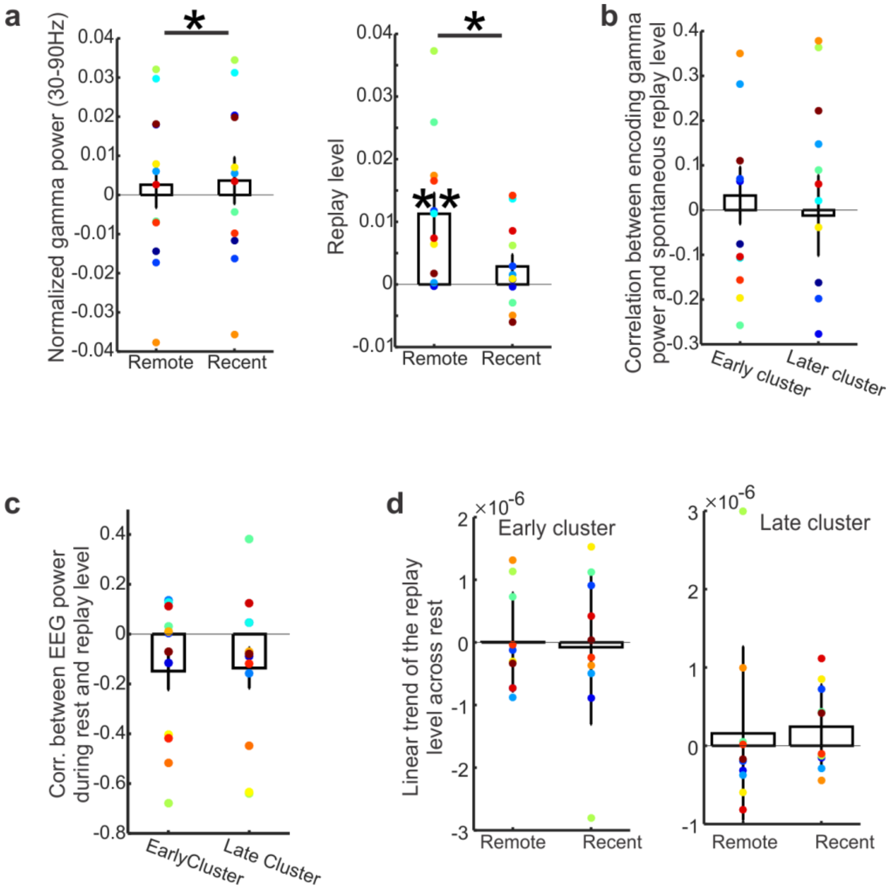

Supplementary Fig. 6. Control analyses of spontaneous replay. (a) Gamma power (30-90Hz) during the late encoding cluster after matching between remote and recent items (left panel). The replay levels of remote items were still higher than the replay levels of recent items (right panel). **(b)** EEG power during encoding is not related to replay levels of brain patterns from either the late or the early encoding cluster. **(c)** EEG power during rest is not related to replay levels. **(d)** Replay is not related to temporal proximity between encoding and rest. Each colorful dot indicates one participant. Same colors indicate data from the same participant. Error bars indicate standard error of the mean (SEM); * indicates $p < 0.05$ (paired T-test); ** indicates $p < 0.01$ (one-sample T-test).

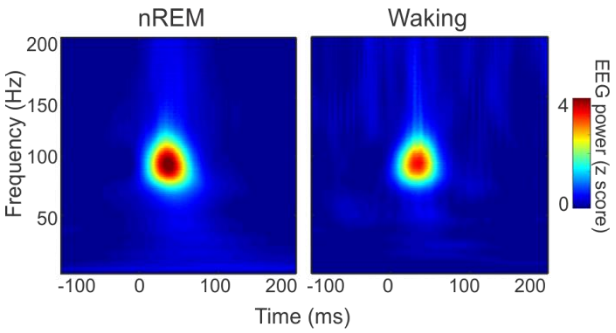

Supplementary Fig. 7. Ripples during different states of vigilance. Ripples with similar properties were observed during nREM sleep and waking state.

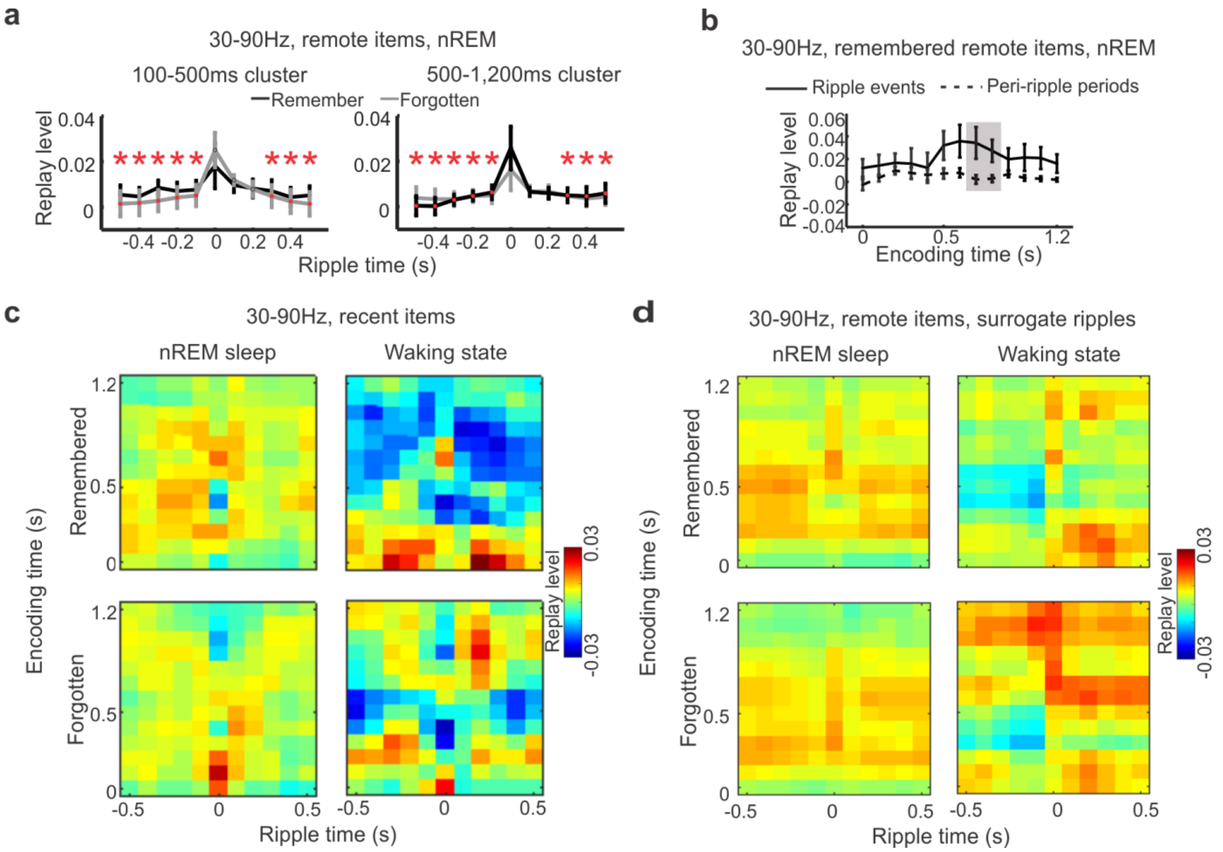

Supplementary Fig. 8. Ripple-triggered replay. (a) Ripple-triggered replay levels of gamma band activity from the early and the late encoding clusters for remembered and forgotten remote items during nREM sleep. Error bars indicate standard error of the mean (SEM); red * indicate peri-ripple periods showing significantly lower replay levels (p<0.05; paired T-test) than ripple events after multiple comparison correction. **(b)** Encoding-time resolved replay levels of gamma band activity during ripple events and peri-ripple periods during nREM sleep. Error bars indicate standard error of the mean (SEM); time bins with gray background indicate encoding time bins showing significantly higher replay levels (p<0.05; paired T-test) during ripple events compared with peri-ripple periods after multiple comparison correction. **(c)** Time resolved replay locked to ripples during different states of vigilance for remembered and forgotten recent items in the gamma band. **(d)** Time resolved replay locked to surrogate ripples during different states of vigilance for remembered and forgotten remote items in the gamma band. Ripple time 0 corresponds to the time point of ripple events and ripple time before and after 0 corresponds to peri-ripple periods.

References

1. Zhang H, Fell J, Staresina BP, Weber B, Elger CE, Axmacher N. Gamma power reductions accompany stimulus-specific representations of dynamic events. *Curr Biol* **25**, 635-640 (2015).

2. Yaffe RB, Kerr MS, Damera S, Sarma SV, Inati SK, Zaghloul KA. Reinstatement of distributed cortical oscillations occurs with precise spatiotemporal dynamics during successful memory retrieval. *Proc Natl Acad Sci USA* **111**, 18727-18732 (2014).

3. Buzsaki G, Wang XJ. Mechanisms of gamma oscillations. *Annu Rev Neurosci* **35**, 203-225 (2012).

4. Pavlides C, Winson J. Influences of Hippocampal Place Cell Firing in the Awake State on the Activity of These Cells during Subsequent Sleep Episodes. *J Neurosci* **9**, 2907-2918 (1989).

5. Skaggs WE, McNaughton BL. Replay of neuronal firing sequences in rat hippocampus during sleep following spatial experience. *Science* **271**, 1870-1873 (1996).

6. Nadasdy Z, Hirase H, Czurko A, Csicsvari J, Buzsaki G. Replay and time compression of recurring spike sequences in the hippocampus. *J Neurosci* **19**, 9497-9507 (1999).

7. Cheng S. The CRISP theory of hippocampal function in episodic memory. *Front Neural Circuits* **7**, 88 (2013).

8. Eichenbaum H. Memory on time. *Trends Cogn Sci* **17**, 81-88 (2013).

9. Ranganath C, Hsieh LT. The hippocampus: a special place for time. *Proc Natl Acad Sci USA* **1369**, 93-110 (2016).
